# Predictive and Explainable Artificial Intelligence for Neuroimaging Applications

**DOI:** 10.3390/diagnostics14212394

**Published:** 2024-10-27

**Authors:** Sekwang Lee, Kwang-Sig Lee

**Affiliations:** 1 Department of Physical Medicine and Rehabilitation, Anam Hospital, Korea University College of Medicine, Seoul 02841, Republic of Korea; 2 AI Center, Anam Hospital, Korea University College of Medicine, Seoul 02841, Republic of Korea

**Keywords:** neuroimaging, predictive artificial intelligence, explainable artificial intelligence

## Abstract

Background: The aim of this review is to highlight the new advance of predictive and explainable artificial intelligence for neuroimaging applications. Methods: Data came from 30 original studies in PubMed with the following search terms: “neuroimaging” (title) together with “machine learning” (title) or ”deep learning” (title). The 30 original studies were eligible according to the following criteria: the participants with the dependent variable of brain image or associated disease; the interventions/comparisons of artificial intelligence; the outcomes of accuracy, the area under the curve (AUC), and/or variable importance; the publication year of 2019 or later; and the publication language of English. Results: The performance outcomes reported were within 58–96 for accuracy (%), 66–97 for sensitivity (%), 76–98 for specificity (%), and 70–98 for the AUC (%). The support vector machine and the convolutional neural network registered the best performance (AUC 98%) for the classifications of low- vs. high-grade glioma and brain conditions, respectively. Likewise, the random forest delivered the best performance (root mean square error 1) for the regression of brain conditions. The following factors were discovered to be major predictors of brain image or associated disease: (demographic) age, education, sex; (health-related) alpha desynchronization, Alzheimer’s disease stage, CD4, depression, distress, mild behavioral impairment, RNA sequencing; (neuroimaging) abnormal amyloid-β, amplitude of low-frequency fluctuation, cortical thickness, functional connectivity, fractal dimension measure, gray matter volume, left amygdala activity, left hippocampal volume, plasma neurofilament light, right cerebellum, regional homogeneity, right middle occipital gyrus, surface area, sub-cortical volume. Conclusion: Predictive and explainable artificial intelligence provide an effective, non-invasive decision support system for neuroimaging applications.

## 1. Introduction

### 1.1. Brain Disease

Brain disease represents a significant contributor to global disease burden [1,2,3]. In 2021, it was estimated that over three billion people globally were affected by neurological conditions [3]. Premature death and disability (disability-adjusted life years, DALYs) from neurological conditions has grown by 18% since 1990. More than 80% of this burden comes from low- and middle-income countries. Furthermore, there is considerable variation in access to treatment, i.e., there are almost 70 times more neurological professionals per 100,000 people in high-income countries compared to low- and middle-income countries. Stroke, neonatal encephalopathy, migraine, dementia, and diabetic neuropathy, as well as meningitis, epilepsy, neurological complications from preterm birth, autism spectrum disorder, and nervous system cancers, were the top 10 neurological conditions in 2021. The burden of brain disease is greater in men compared to women in general. However, there exist certain exceptions of female dominance, including migraine and dementia [3]. There are many types of brain disease, e.g., autoimmune brain diseases, epilepsy, infections, mental illness (i.e., anxiety, bipolar disorder, depression, post-traumatic stress disorder, schizophrenia), neurodegenerative brain diseases (Alzheimer’s disease, Parkinson’s disease, amyotrophic lateral sclerosis), neurodevelopmental disorders (attention deficit hyperactivity disorder, autism spectrum disorder, dyslexia), stroke, traumatic brain injuries, and tumors [4,5,6].

Autoimmune brain diseases are characterized by the body’s immune system attacking a part of the brain, which it identifies as an invader. Epilepsy is defined as a tendency to experience seizures, which are characterized by electrical disturbances in the brain. These seizures typically disrupt consciousness and manifest as convulsions, which are uncontrolled muscle movements. Infections occur when various types of pathogens invade the brain or its protective coverings. Mental, behavioral, and emotional disorders have the potential to impair an individual’s quality of life and their capacity to function effectively. The principal categories are as follows: Anxiety, Bipolar disorder, Depression, Post-traumatic stress disorder (PTSD), and Schizophrenia. The accumulation of abnormal proteins in the brain is a common underlying cause of neurodegenerative disorders. These include conditions such as Alzheimer’s disease, Parkinson’s disease, and ALS (amyotrophic lateral sclerosis), among numerous others. Neurodevelopmental disorders impact the growth and development of the brain, with care typically provided by pediatric neurologists. Medical geneticists may ascertain the likelihood of an inherited disorder. In the event that a genetic predisposition is identified, family counselling is provided. A considerable number of neurodevelopmental disorders exist, including Attention deficit hyperactivity disorder (ADHD), Autism spectrum disorder, and Dyslexia. A stroke is defined as the obstruction or rupture of a cerebral blood vessel, which results in the interruption of cerebral blood flow and subsequent injury to the brain parenchyma. Traumatic brain injuries encompass a range of conditions, from mild concussions to more severe injuries such as gunshot wounds. Additionally, brain tumors may result from the metastasis of malignant cells from other regions of the body, including the lungs, breasts, and colon. Alternatively, they may develop within the brain tissue itself or its coverings. Astrocytomas are a common type of tumors that originate from the brain itself. A meningioma is a common tumor that develops from the coverings of the brain [6].

### 1.2. Neuroimaging and Artificial Intelligence

The concepts of neuroimaging and artificial intelligence have recently attracted global interest. A brain imaging method can be defined as any experimental technique that allows for the study of the structure or function of the human (or animal) brain, preferably in vivo in the context of the present study [7]. The optimal method should yield precise temporal and spatial localization of cerebral function, structure, or alterations in these properties. The optimal method should involve minimum invasion and maximum replication for treatment monitoring and therapeutic development as well. Structural magnetic resonance imaging (MRI) meets these optimal requirements for structural imaging. However, there is no single optimal technique for functional imaging, even though electroencephalography (EEG), positron emission tomography (PET), and functional magnetic resonance imaging (fMRI) are very popular. EEG and PET have been available for 4 decades or more, whereas functional magnetic resonance imaging (fMRI) is the newest widely used technique. Arguable, PET is the most invasive with the administration of radioisotopes and EEG has poor spatial mapping properties. Given these limitations, fMRI has become as the most common functional brain-mapping approach [7].

On the other hand, artificial intelligence can be denoted as “the capability of a machine to imitate intelligent human behavior” (the Merriam–Webster dictionary). As a division of artificial intelligence, machine learning can be considered to be “extracting knowledge from large amounts of data” [8]. Popular machine learning approaches are the decision tree, the naïve Bayesian, the random forest, the support vector machine, and the neural network. (See more detailed explanations for [8,9].) In particular, a random forest is a group of decision trees that collectively makes a majority decision regarding the dependent variable, a process known as “bootstrap aggregation.” For the purposes of this discussion, we will consider a random forest comprising 1000 decision trees. For the purposes of this discussion, we shall assume that the original data set comprises 10,000 participants. Subsequently, the training and testing of this random forest is conducted in two stages. Initially, new data comprising 10,000 participants is generated through random sampling with replacement, upon which a decision tree is constructed. In this process, some participants from the original data set are excluded from the new data set, and these remaining participants are referred to as the “out-of-bag” data set. This process is repeated 1000 times, resulting in the creation of 1000 new data sets, 1000 decision trees, and 1000 out-of-bag data sets. Secondly, the 1000 decision trees make predictions regarding the dependent variable for each participant in the out-of-bag data. Then, the majority vote is taken as the final prediction for that participant, and the out-of-bag error is derived as the proportion of incorrect votes for all participants in the out-of-bag data sets. An artificial neural network is a network of neurons (information units) based on a set of weights. Typically, it has one input layer, one or more intermediate layers, and one output layer [9]. A deep neural network is an artificial neural network having a large number of intermediate layers, with the number of layers often being in the range of 5 to 1000 [9].

The current research paradigm has a limited scope in terms of the predictors considered for the early diagnosis of disease. This is due to the use of logistic regression, which assumes a rather unrealistic condition of *ceteris paribus*, or “all other variables remaining constant”. In light of the aforementioned limitations, the literature on the early diagnosis of disease is increasingly turning to artificial intelligence. This includes studies on arrhythmia [10], birth outcome [11], cancer [12,13], comorbidity [14], depression [15], liver transplantation [16], menopause [17,18], and temporomandibular disease [19]. It is not constrained by the unrealistic assumption of “all the other variables staying constant.” Furthermore, the concept of explainable artificial intelligence is currently experiencing a surge in popularity. The term “explainable artificial intelligence” is defined as “artificial intelligence to identify major predictors of the dependent variable”. At this point in time, three popular approaches to explainable artificial intelligence have been identified: namely, random forest impurity importance, random forest permutation importance, and machine learning permutation importance [20]. The random forest impurity importance metric quantifies the reduction in node impurity resulting from the creation of a branch on a specific predictor. The random forest permutation importance metric quantifies the overall reduction in accuracy resulting from the random permutation of data on a given predictor. An extension of random forest permutation importance, machine learning accuracy importance calculates the decrease in accuracy resulting from the permutation of data on the predictor [20]. However, more study is needed on the review of artificial intelligence for neuroimaging applications. This study reviews the recent progress of predictive and explainable artificial intelligence for neuroimaging applications.

## 2. Methods

**Figure 1:**
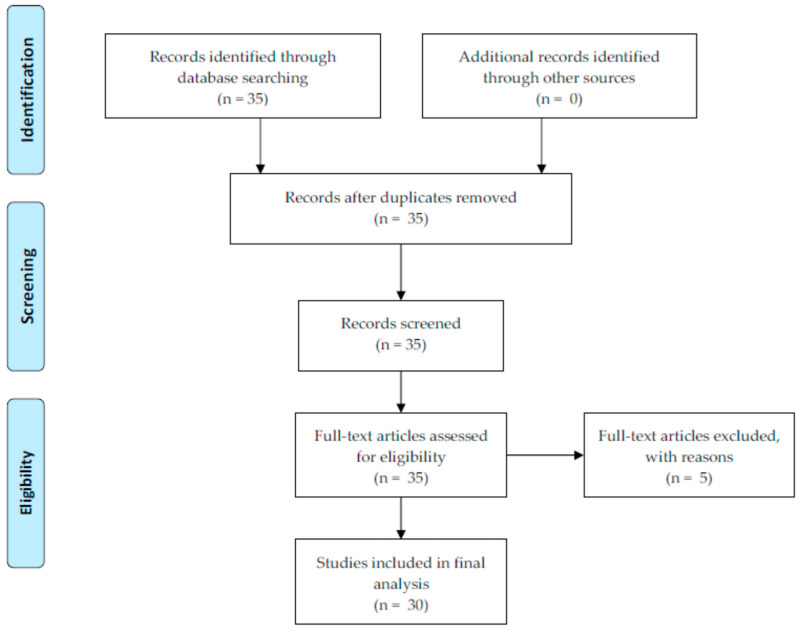
Flow diagram.

Figure 1 shows the flow diagram of this study as a modified version of Preferred Reporting Items for Systematic Reviews and Meta-Analyses. The source of data was 30 original studies in PubMed. The search terms were “neuroimaging” (title) together with “machine learning” (title) or “deep learning” (title). The eligibility criteria were the participants with the dependent variable of brain image or associated disease, the interventions/comparisons of artificial intelligence, the outcomes of accuracy, the AUC and/or variable importance, the publication year of 2019 or later, and the publication language of English. Opinions, reports, and reviews were excluded. The following summary measures were adopted: (1) sample size (participants), baseline vs. innovation artificial intelligence methods (comparisons vs. interventions), dependent variable (participants), task type; (2) baseline vs. innovation performance outcomes; (3) major demographic, health-related, and neuroimaging predictors. Here, accuracy denotes the proportion of correct predictions over all observations. The area under the curve (AUC) represents the area under the plot of the true positive rate (sensitivity) against the false positive rate (1-specificity) at various threshold settings. The AUC is a major performance criterion in this study, given that it accommodates sensitivity and specificity.

## 3. Results

### 3.1. Summary

The summary of the review for the 30 original studies [21,22,23,24,25,26,27,28,29,30,31,32,33,34,35,36,37,38,39,40,41,42,43,44,45,46,47,48,49,50] is presented in Table 1, Table 2, Table 3 and Table 4. The “Study” column in the tables denotes the reference numbers of the 30 original studies. Also, abbreviations are listed in Table 5. The tables include (1) sample size, baseline vs. innovation artificial intelligence methods, dependent variable and task type (Table 1); (2) baseline vs. innovation performance outcomes (Table 2); (3) major demographic, health-related, and neuroimaging predictors (Table 3); (4) cross validation and major control variable (Table 4). The ranges of performance measures were reported to be 58–96 for accuracy (%), 66–97 for sensitivity (%), 76–98 for specificity (%), and 70–98 for the AUC (%). The support vector machine and the convolutional neural network registered the best performance (AUC 98%) for the classifications of low- vs. high-grade glioma [28] and brain image properties [44], respectively. Similarly, the random forest delivered the best performance (root mean square error 1) for the regression of brain image properties [43]. The following factors were discovered to be major predictors of brain image or associated disease: (demographic) age, education, sex; (health-related) alpha desynchronization, Alzheimer’s disease stage, CD4, depression, distress, mild behavioral impairment, RNA sequencing; (neuroimaging) abnormal amyloid-β, amplitude of low-frequency fluctuation, cortical thickness, functional connectivity, fractal dimension measure, gray matter volume, left amygdala activity, left hippocampal volume, plasma neuro-filament light, right cerebellum, regional homogeneity, right middle occipital gyrus, surface area, sub-cortical volume. Finally, 22 original studies included cross validation, and 14 studies matched control and experimental groups in age, sex, and/or education (defined as “major control variables” in Table 4). The differences between the control and experimental groups in terms of the major control variables were statistically insignificant in the 14 studies. Predictive and explainable artificial intelligence provide an effective, non-invasive decision support system for neuroimaging applications. However, artificial intelligence is a data-driven approach, and more research is needed for more general conclusions given that the findings of this study above were based on the 30 original studies published in 2019 or later.

### 3.2. Predictive Artificial Intelligence

This section summarizes original studies, which highlight the strengths of predictive artificial intelligence with the best performance metrics for neuroimaging applications [28,43,44]. As addressed above, the support vector machine registered the best performance (AUC 98%) for the classifications of low- vs. high-grade glioma in one study [28]. MRI data on texture and fractal dimension measures came from 28 glioma patients enrolled in a national medical institute. The dependent variable was the grade of glioma with 0 (low) vs. 1 (high). The independent variables were 25 texture and 15 fractal dimension indicators. The accuracy, sensitivity, specificity, and AUC of the support vector machine were 93%, 97%, 98%, and 98% for the general structure of the enhanced tumor, respectively. These best results were followed by those of the boundary of the whole tumor, i.e., the accuracy, sensitivity, specificity, and area under the curve of 83%, 100%, 60%, and 80%. These findings of multivariable machine learning were consistent with their univariate counterparts. The fractal dimension measures of high-grade glioma were significantly greater than those of low-grade glioma: 1.221 vs. 1.626 for the general structure of the enhanced tumor (*p* < 0.0001); 0.923 vs. 0.940 for the boundary of the whole tumor (*p* = 0.0105). This study suggests that the separate examination of the whole tumor and its elements is expected to present important insights regarding predictive artificial intelligence for neuroimaging applications.

Likewise, the convolutional neural network presented the best AUC of 98% for the classifications of brain conditions [44]. The source of MRI data was 59 study participants. The outcome variables are somatic pain and social rejection. The input variables were brain networks such as visual, somatomotor, dorsal attention, salience network, limbic, frontoparietal, and default. The convolutional neural network was a little better than the support vector machine as predictive artificial intelligence, i.e., 96%, 96%, 955, and 98% vs. 92%, 94%, 91%, and 97% in terms of accuracy, sensitivity, specificity, and AUC. In a similar context, the random forest delivered the best performance (root mean square error less than 1) for the regression of brain conditions [43]. Data consisted of 400 study participants. The dependent variable was cognitive ability (measured by the Global Cognitive Assessment Task), and the independent variable was the gray matter volume. The random forest outperformed the elastic net and ridge regression in terms of the root mean square error less than 1. The findings above demonstrate that the best predictive artificial intelligence models for neuroimaging applications vary depending on different outcome variables and different input variables. Little study was done, and more analysis is needed regarding which models serve as the best predictive artificial intelligence for varying brain conditions with varying analytic tasks.

### 3.3. Explainable Artificial Intelligence

This section summarizes original studies, which request due attention to the strengths of explainable artificial intelligence with multiple-domain data for brain disease applications [27,29,31,41]. The aim of a recent study was to develop explainable artificial intelligence for the classification of frailty in Human Immunodeficiency Virus (HIV) patients [27]. The source of MRI data was 105 study participants enrolled in a university medical center. The outcome variable was frailty in HIV patients. The input variables were demographic (sex), health-related (depression, CD4), and neuroimaging predictors. The sensitivity and F1 score of boosting were 66% and 71%, correspondingly. Based on boosting permutation variable importance, the top five predictors were reduced cerebral blood flow in the right pallidum region, reduced cerebral blood flow in the left occipital region, lower psychomotor performance, reduced volume of the right pericalcarine region, and lower resting-state functional connectivity between the frontal parietal and ventral attention networks. Likewise, another study attempted to highlight the strengths of boosting as explainable artificial intelligence for the regression of brain age [29]. Data consisted of 22,661 study participants enrolled in national projects. The dependent variable was brain age, and the independent variables were demographic (sex), health-related (Alzheimer’s disease stage), and neuroimaging (Abnormal Amyloid-β, APOE-ε4, and plasma neurofilament light). The root mean square error of boosting was 4.

In a similar vein, the purpose of a recent study centered on developing explainable artificial intelligence for the classification of glioblastoma survival [31]. The source of MRI data was 133 study participants enrolled in a university medical center. The outcome variable was glioblastoma survival. The input variables were demographic (age, sex) and neuroimaging (cortical thickness, functional connectivity). The accuracy of the artificial neural network was 91%. According to artificial neural network permutation variable importance, the top five predictors were functional connectivity for distance correlation 10, Bankstss cortex, age, sex, and functional connectivity for distance correlation 11. The success of these machine learning studies was extended to its deep learning counterpart, which endeavored to demonstrate the strengths of the residual convolutional neural network as explainable artificial intelligence for the classification of motor performance in stroke [41]. Data consisted of 41 study participants enrolled in previous studies. The dependent variable was motor performance in stroke, and the independent variables were demographic (age, sex) and neuroimaging (axial diffusivity, fractional anisotropy, mean diffusivity, radial diffusivity, white matter, gray matter). The performance measures of the support vector machine and the residual convolutional neural network were similar to each other, i.e., 91% and 91% vs. 92% and 92% in terms of accuracy and AUC.

## 4. Discussion

The existing literature on predictive and explainable artificial intelligence for neuroimaging applications has some limitations. Firstly, a majority of the studies reviewed here were characterized by single-centre data with relatively small sample sizes. The utilization of multi-centre data will facilitate further advancements in this field of research. Indeed, more analysis is needed regarding the effect of the sample size on model performance. One study reviewed here [33] made a rare attempt in this direction. As the sample size increased from 100 to 10,000, an accuracy gap between machine learning (support vector machine) and deep learning (convolutional neural network-Alex) increased to 7% (51% vs. 58%) for the prediction of 10 brain age groups in this study. But more examination is needed on this topic, given that both machine learning and deep learning registered low performance and their performance difference was not very large in this study. Secondly, the accuracies of some studies reviewed here (58%) may not yet meet the standards required for use as diagnostic tests. In addition, only seven studies reviewed here used test sets, and these test sets came from internal sources. Despite these limitations, these studies were included in this review, given that the further advance of predictive and explainable artificial intelligence for neuroimaging applications is not possible without trials and errors.

Thirdly, three common methods of explainable artificial intelligence (machine learning permutation importance, random forest permutation importance, and random forest impurity importance) may yield different outcomes on some occasions. The random forest impurity importance shows more variation from the categorization of variables. However, the random forest has a special quality of including sequential information, and this special quality is more apparent with the random forest impurity importance. In this context, extensive comparison for the three methods of explainable artificial intelligence would be a major achievement for this line of research. Fourthly, other types of explainable artificial intelligence and trade-offs between predictive power and explainable power were beyond the scope of this review, e.g., local interpretable model-agnostic explanations (LIME) [51]. Fifthly, 22 studies reviewed here employed cross validation, but only eight studies reported performance measures over each subset [26], their standard deviations across all subsets [36], or their confidence intervals across all subsets [41,44,45,46,47,48]. Especially, this was a significant drawback for five out of nine studies with deep learning models with cross validation. In other words, there can be found certain risks of detection, attrition, and reporting biases. This issue requests much more attention for the future studies on this topic.

Sixthly, hyper-parameter tuning was either absent or basic in the studies reviewed here. One possible explanation is that neuroimaging investigation itself requires significant time and energy besides hyper-parameter tuning. In spite of this reality, it is still a valid suggestion that advanced hyper-parameter tuning is expected to bring significant improvement in the performance of predictive and explainable artificial intelligence for neuroimaging applications. One plausible approach of advanced hyper-parameter tuning is the policy gradient approach [52] (within reinforcement learning to be addressed below). Here, the policy gradient can be defined as “the change of action to maximize the reward”, e.g., the change of hyper-parameter selection to maximize the performance of predictive and explainable artificial intelligence for neuroimaging applications. In other words, the policy gradient approach can be denoted as “systematic hyper-parameter selection”, i.e., finding the optimal values of hyper-parameters based on performance measures and major control variables [52]. These new approaches would expand the boundary of knowledge by a great extent. Seventhly, experts in the field of artificial intelligence focus on the performance of predictive and explainable artificial intelligence as the best indicator of study quality. We followed this convention.

Indeed, some suggestions for this line of research are presented here. Firstly, synthesizing various forms of explainable artificial intelligence with various forms of data in the field of brain disease would represent a significant advancement in the field. An increasing amount of artificial intelligence research is synthesizing genetic, image, and numeric methods for disease diagnosis, treatment, and management. This new approach is called “wide and deep learning”, and it includes a great variety of multi-input multi-output combinations. A recent study [53] serves as a good example, given that it presents a glaucoma prediction system combing convolutional neural networks and their recurrent neural network counterparts. Here, the former network draws key image characteristics from multiple image inputs, and the latter part predicts glaucoma results from the course of the key image characteristics over time. In a convolutional neural network, filters look around input data and detect certain characteristics based on their convolution operations. (This predicts the status of normal versus disease.) In a recurrent neural network, output in the present is determined in a recurrent pattern by input in the present and memory in the past (which is called “the hidden state in the past”) [8,9]. There is a paucity of literature on this topic, and further investigation is required to gain insight into the integration of diverse forms of explainable artificial intelligence for diverse data types in the context of brain disease applications.

Secondly, little examination has been done, and more investigation is needed on reinforcement learning. Reinforcement learning has three key components: the environment bringing a series of rewards, an agent taking a series of actions to maximize the cumulative reward, and the environment transitioning to the next period with given transition probabilities [54]. Here, artificial intelligence (e.g., Alpha-Go) begins in a manner similar to that of a human player, taking a series of actions and maximizing the cumulative reward (chance of victory) from the limited information available in limited periods only. Then, it is capable of surpassing the performance of the best human player ever, due to the immense power of big data covering all human players to date [54]. The popularity of reinforcement learning in finance and health can be attributed to its ability to achieve excellent results without the need for unrealistic assumptions, while offering a superior performance compared to conventional statistical models [55,56]. Nevertheless, there is a paucity of literature on the subject, and further investigation is required in order to gain a deeper understanding of explainable reinforcement learning. A recent review indicates that only a few studies have addressed this issue. These studies have employed simplified models with straightforward interpretations but have demonstrated inadequate performance and have given insufficient consideration to the psychological and social factors underlying optimization processes [57].

Thirdly, rigorous qualitative evaluation approaches need to be developed regarding systematic reviews of predictive and explainable artificial Intelligence for neuroimaging applications. The Enhancing the Quality and Transparency of Health Research Network recommends neuroimaging meta-analysis to include the following information: research question; eligibility and exclusion criteria; flow diagram; experimental characteristics such as sample size (participants), baseline vs. innovation methods (comparisons vs. interventions), dependent variable (participants), task type, baseline vs. innovation performance outcomes, and participant characteristics [58,59]. This study followed this recommendation with the following summary measures: research question (p. 003); eligibility and exclusion criteria (pp. 003–004); flow diagram (Figure 1); experimental characteristics such as (1) sample size, baseline vs. innovation artificial intelligence methods, dependent variable, and task type (Table 1), (2) baseline vs. innovation performance outcomes (Table 2), (3) major demographic, health-related, and neuroimaging predictors (Table 3), and (4) cross validation and major control variable (Table 4). However, more systematic qualitative evaluation methods can be designed, and this new guideline is expected to improve the reliability of reviews for predictive and explainable artificial intelligence for neuroimaging applications much more.

## 5. Conclusions

In summary, this study reviewed the recent progress of predictive and explainable artificial intelligence for neuroimaging applications. The ranges of performance measures were reported to be 58–96 for accuracy (%), 66–97 for sensitivity (%),76–98 for specificity (%), and 70–98 for the AUC (%). The support vector machine and the convolutional neural network registered the best performance (AUC 98%) for the classifications of low- vs. high-grade glioma and brain conditions, respectively. Similarly, the random forest delivered the best performance (root mean square error 1) for the regression of brain conditions. The following factors were discovered to be major predictors of brain image or associated disease: (demographic) age, education, sex; (health-related) alpha desynchronization, Alzheimer’s disease stage, CD4, depression, distress, mild behavioral impairment, RNA sequencing; (neuroimaging) abnormal amyloid-β, amplitude of low-frequency fluctuation, cortical thickness, functional connectivity, fractal dimension measure, gray matter volume, left amygdala activity, left hippocampal volume, plasma neuro-filament light, right cerebellum, regional homogeneity, right middle occipital gyrus, surface area, sub-cortical volume. Combining various types of explainable artificial intelligence with various types of information in the field of brain disease would bring significant progress in the field. Little research has been done, and more study is needed on reinforcement learning. In spite of these limitations, predictive and explainable artificial intelligence provide an effective, non-invasive decision support system for neuroimaging applications.

## Figures and Tables

**Table 1:** Summary—Sample Size Method and Dependent Variable.

Study	Sample Size	Method-Baseline	Method-Innovation	Dependent Variable	Type
21	109		Global Signal Regression	Schizophrenia	Classification
22	915	CNN-Dense	CNN-Dense SDA	ASD	Classification
23	500		Unet	Brain Image	Generation
24	78		LASSO	Suicidal Thought	Regression
25	387		RF	Anxiety in MDD	Classification
26	638		CNN-VGG	Four Brain Age Groups	Classification
27	105		Boosting	Frailty in HIV	Classification
28	42		SVM	Glioma	Classification
29	22,661		Boosting	Brain Age	Regression
30	70		SVM	Chronic Sciatica	Classification
31	133		ANN	Glioblastoma Survival	Classification
32	19		LDA	Opiate Addiction	Correlation
33	10,000	LDA LR SVM *	CNN-Alex	10 Brain Age Groups	Classification
34	160		CNN-FastSurfer	Brain Condition	Classification
35	84		SVM	Insomnia in Hemodialysis	Classification
36	3000		CNN-Dense	Dementia	Classification
37	47		CNN	Pediatric Brain Tissues	Generation
38	206		DT LR RF * SVM	ASD and Schizophrenia	Classification
39	500		Unet	Brain Vascular	Generation
40	103	ANN Uni-Modal	ANN Multi-Modal	Schizophrenia	Classification
41	154	DT KN NB RF SVM *	CNN-Residual	Post-Stroke Motor	Classification
42	81		CNN-Residual	Brain Image	Generation
43	400		EN RF * RR	Brain Condition	Regression
44	59	SVM	CNN	Brain Condition	Classification
45	341		DT	Dementia	Classification
46	688		SVM	Schizophrenia	Classification
47	172	DT * KN LR SVM	Graph Neural Network	Schizophrenia	Classification
48	180		CNN	Brain Image	Generation
49	956		CNN	Psychosis	Classification
50	1130		CNN-Alex	Parkinson’s Disease	Classification

Note: * Best Model.

**Table 2:** Summary—Model Performance.

Study	Performance-Baseline				Performance-Comparison			
	Acc	Sen	Spe	AUC **	Acc	Sen	Spe	AUC **
21					83	69	94	85
22				86				93
23								70
24								NA
25								80
26								73
27						66		71
28					93	97	98	98
29								4
30					90			
31					91			
32								83
33	51				58			
34					96	96		
35					82			82
36					95	96	95	95
37								90
38					76			83
39								93
40	55			69	71			92
41	91			91	92			92
42								NA
43								1
44	92	94	91	97	96	96	95	98
45					84			86
46					60		84	
47	78	83	72	79	80	84	76	80
48								97
49					70			
50					96			95
Min					58	66	76	70
Max					96	97	98	98

Note: ** Correlation (Correlation) R-Square (Regression) or Structural Similarity Index Measure (Generation). 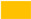 F1 (Classification) Root Mean Square Error (Regression) or R-Square (Generation).

**Table 3:** Summary—Major Predictor.

Study	Predictor Demographic	Predictor Health	Predictor Neuroimaging
21			
22			
23			
24	Age Education	Depression Distress	LAA
25			GMV ALFF RH FC
26			FC
27	Sex	Depression CD4	Neuroimaging
28			FDM
29	Sex	Alzheimer’s Disease Stage	AAB APOE-ε4 PNL
30			FC ALFF SA Combination
31	Age Sex		CT FC
32		Alpha Desynchronization	FC
33			
34			
35			ALFF RMCG RC
36			
37			
38			CT SCV
39			
40		RNA Sequencing	Neuroimaging
41	Age Sex		Neuroimaging
42			GMV
43			GMV
44			
45		Mild Behavioral Impairment	LHV
46			
47			
48			
49			GMV CT
50			

**Table 4:** Summary—Cross Validation and Major Control Variable.

Study	Sample Size	Training	Validation	Test	N-Fold CV *	Major Control Variable
21	1029	799	89	141		Age Sex
22	915	488	244	183	3	Sex
23	500	500	500			
24	78	70	8		10	Emotion Physiology
25	387	348	39		10	
26	638	408	102	128	5	Age Sex
27	105	84	21		5	
28	42	23	6	13	5	
29	24,975	20,395	2266	2314	10	Age
30	16,100	15,870	230		70	Age Sex Education Occupation
31	133	132	1		133	
32	19	19				Age Education IQ
33	12,314	10,000	1157	1157		Age Gender
34	160	140	20			Age Gender
35	84	83	1		84	Age Sex Education
36	3000	2400	600		5	
37	47	47	47			
38	206	165	41		5	Age Sex
39	500	500	500			
40	103	83	20		5	Age Sex
41	154	124	30		5	
42	81	81	81			
43	400	360	40		10	
44	59	52	7		8	
45	340	306	34		10	Age Education
46	688	619	69		10	Age Sex
47	172	155	17		10	
48	180	128	32	20	5	
49	956	860	96		10	
50	1130	1020	110		10	

Note: * N-Fold Cross Validation for Training-Validation Sets. 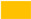 Leave-One-Out Cross Validation (Every Single Element Serves as the Validation Set).

**Table 5:** Abbreviation.

*Method*	
ANN	Artificial Neural Network
CNN	Convolutional Neural Network
DT	Decision Tree
EN	Elastic Net
KN	K-Nearest Neighbor
LASSO	Least Absolute Shrinkage and Selection Operator
LDA	Linear Discriminate Analysis
LR	Logistic Regression
NB	Naïve Bayes
RF	Random Forest
RR	Ridge Regression
SDA	Supervised Domain Adaptation
SVM	Support Vector Machine
VGG	Virtual Geometry Group
*Dependent Variable*	
ASC	Attenuation-Scatter Correction
ASD	Autism Spectrum Disorder
HIV	Human Immunodeficiency Virus
MDD	Major Depressive Disorder
*Model Performance*	
Acc	Accuracy
Sen	Sensitivity
Spe	Specificity
AUC	Area Under the Curve
*Predictor Neuroimaging*	
AAB	Abnormal Amyloid-β
ALFF	Amplitude of Low-Frequency Fluctuation
CT	Cortical Thickness
FC	Functional Connectivity
FDM	Fractal Dimension Measure
GMV	Gray Matter Volume
LAA	Left Amygdala Activity
LHV	Left Hippocampal Volume
PNL	Plasma Neurofilament Light
RC	Right Cerebellum
RH	Regional Homogeneity
RMCG	Right Middle Occipital Gyrus
SA	Surface Area
SCV	Sub-Cortical Volume
